# Inhibition of Exo70, an Exocyst Complex Component, Enhances mRNA Delivery Efficiency of Lipid Nanoparticle

**DOI:** 10.3390/pharmaceutics18060650

**Published:** 2026-05-25

**Authors:** Minki Ha, Seok-Beom Yong

**Affiliations:** Department of Medical and Biological Sciences, The Catholic University of Korea, Bucheon 14662, Gyeonggi-do, Republic of Korea

**Keywords:** LNP, exocytosis, endosomal recycling, Exo70

## Abstract

**Background/Objectives:** Lipid nanoparticles (LNPs) are actively being studied as therapeutics and vaccines for various diseases. While LNPs can deliver nucleic acids, their efficiency is limited by the multi-step pathways involved in intracellular trafficking. Crucially, endosomal recycling-driven exocytosis acts as a major problem, rerouting LNPs away from the cytosol and thereby preventing efficient nucleic acid release. Upon entering the cell, LNPs are frequently expelled via endosomal recycling before delivering nucleic acids to cytosol. Previous studies reported that inhibition or deletion of Exo70, a component of the exocyst complex, leads to the accumulation of endosomes because of preventing endosomal recycling. In this study, we investigate the impact of Exo70 inhibition by endosidin-2 (ES-2), an Exo70 inhibitor, on LNP delivery efficiency. **Methods:** SM-102, cholesterol, DMG-PEG, and DSPC were dissolved in ethanol, while mRNA was dissolved in an aqueous phase to formulate LNPs. Co-treatment of ES-2 with LNPs was performed to evaluate its effect on mRNA delivery, and the resulting delivery efficiency was assessed both in vitro and in vivo. **Results:** Co-treatment of ES-2 with LNPs significantly enhanced mRNA delivery efficiency, resulting in up to a 4.06-fold increase in vitro and a 3.63-fold increase in vivo. **Conclusions:** Our findings demonstrate that suppression of Exo70 significantly enhances the mRNA delivery efficiency of LNPs, and this strategy could be applied for the development of mRNA therapeutics.

## 1. Introduction

Lipid nanoparticles (LNPs) have been widely used as delivery carriers for mRNA vaccines, most notably for SARS-CoV-2 during the COVID-19 pandemic [1,2]. LNP-based delivery systems have been employed in several clinically approved therapies, including the Pfizer-BioNTech (BNT162b2) and Moderna (mRNA-1273) COVID-19 vaccines, as well as the FDA-approved siRNA therapeutic patisiran (Onpattro) [1,3]. In general, LNPs are composed of four lipid components: ionizable lipid, cholesterol, helper lipid, and PEG-lipid. This formulation not only protects nucleic acid but also enables efficient delivery of mRNA into target cells [4].

LNPs enter cells via endocytosis and move through the early endosome, which is then converted into a late endosome during endosomal maturation [5]. During endosomal maturation, the progressive acidification of the endosomal lumen induces protonation of ionizable lipids within LNPs, resulting in the acquisition of a positive charge that promotes interactions with the negatively charged endosomal membrane [6,7,8]. Upon interaction with the endosomal membrane and subsequent endosomal escape, LNPs release their genetic cargos into the cytosol [9]. The Rab7-exposed late endosome fuses with the lysosome through interactions with cytosolic SNARE complexes, leading to its transition into an endolysosome [10,11]. Within this compartment, LNPs that fail endosomal escape are eventually degraded by lysosomal acid hydrolases [5,12].

One of the current problems with LNP is the low delivery efficiency, resulting in only about 1–4% of the total administered nucleic acid entering the cell [12,13,14]. Several factors have been reported to contribute to the low delivery efficiency of LNPs, including lysosomal degradation [5,12,15], endosomal recycling [5,15,16], and inhibition of mRNA expression due to immune stimulation of LNPs [17,18,19]. Among these, endosomal recycling is a mechanism in which LNPs are exported from cells through exocytosis before they enter the late endosome for endosomal escape [2,20,21]. Furthermore, it has been revealed that the physical properties and lipid composition of LNPs are affected through exocytosis [16,22].

Previously, deletion of NPC1, a regulator of endosomal recycling, in mouse embryonic fibroblasts (MEFs) significantly increased LNP-mediated siRNA gene silencing, supporting the concept that impaired endosomal recycling prolongs endosomal retention and facilitates more efficient cytosolic delivery of siRNA [15]. Furthermore, in a recent study, co-treatment of LNPs with small-molecule inhibitors of the endocytic recycling pathway improved the mRNA delivery efficiency of LNPs, which is mediated by inhibition of ARF6 or Annexin A6, thereby promoting cytosolic mRNA release [23].

**Figure 1:**
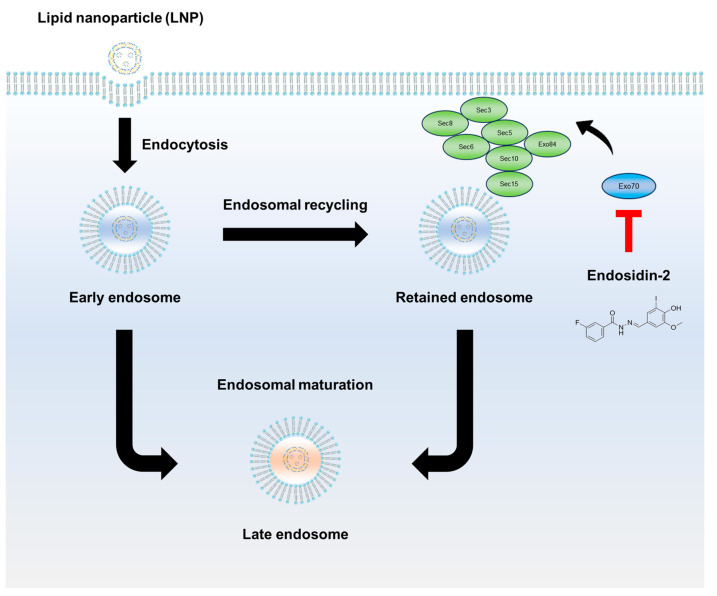
Schematic illustration of ES-2 co-treatment strategy. Exo70 inhibition by ES-2 disrupts exocyst-mediated vesicle trafficking to the plasma membrane, suppressing exocytosis and consequently impairing endosomal recycling. This leads to prolonged endosomal retention of LNPs within cells, ultimately enhancing cytosolic mRNA delivery.

Exo70 is a component of the exocyst complex in mammalian cells that localizes to the plasma membrane and endosomal compartments, where it mediates vesicle tethering during exocytosis [24,25,26]. Exo70 is targeted by the small-molecule inhibitor endosidin-2 (ES-2), which inhibits exocytosis through binding to the Exo70 protein and was originally identified through plant-based small-molecule screening as a trafficking inhibitor [27]. Given that its inhibitory mechanism is conserved in mammals, ES-2 is widely utilized as a chemical agent in studies related to endosomes and exocytosis [27,28,29,30]. In previous studies, intracellular accumulation of late endosomes was observed upon knockdown or pharmacological inhibition of Exo70, indicating impaired endosomal trafficking to the plasma membrane and defective exocytosis [30,31]. In another study, shRNA-mediated Exo70 knockdown or ES-2 treatment increased the number of intracellular CD63-positive multivesicular endosomes (MVEs), a specialized subtype of late endosomes, without affecting their size [30]. Additionally, treatment of ES-2 in HeLa cells inhibited transferrin recycling [27] and co-administration of cisplatin and ES-2 reduced the release of cell-delivered cisplatin, thereby enhancing the anticancer effect of cisplatin in vivo [28]. To the best of our knowledge, effect of Exo70-inhibition on mRNA/LNP delivery has not been studied yet. To this end, we targeted Exo70 and evaluated its impact on mRNA/LNP delivery efficiency, showing that co-treatment of LNP with ES-2 enhances mRNA expression in both in vitro and in vivo (Figure 1).

## 2. Materials and Methods

### 2.1. ES-2, mRNA, and LNP Preparation

Endosidin-2 (ES-2) was purchased from MedChemExpress (Monmouth Junction, NJ, USA) and prepared as a 100 mM stock solution in DMSO. Firefly luciferase mRNA (mLuc) was purchased from TriLink (San Diego, CA, USA). SM-102 was purchased from BroadPharm (San Diego, CA, USA), and DSPC, DMG-PEG2000, and cholesterol were purchased from Avanti Polar Lipids (Alabaster, AL, USA). The lipid phase was prepared by dissolving the SM102, DSPC, DMG-PEG2000, and Cholesterol in ethanol at a molar ratio of 43.5:10:1.5:45 [32]. The aqueous phase was prepared by adding mRNA to a 25 mM acetate buffer (pH 4.5), ensuring that the volume ratio of the lipid phase to the aqueous phase was 1:3 during the microfluidic mixing. LNPs were prepared using the microfluidic mixing device, Nanoassemblr IGNITE (Precision NanoSystems Inc., Vancouver, BC, Canada), with a flow rate of 12 mL/min. The prepared LNP was dialyzed in PBS for approximately 18 h. After dialysis, the LNPs were stored at 4 °C for subsequent use in experiments.

### 2.2. mRNA Encapsulation Efficiency and LNP Characterization

To quantify the amount of mRNA encapsulated in LNPs, a RiboGreen assay kit (Thermo Fisher Scientific Inc., Waltham, MA, USA) was used. LNP samples were diluted 200-fold in TE buffer, either containing 0.5% Triton or without Triton, and incubated at 37 °C for 10 min. The samples were then transferred to a 96-well black plate, and TE buffer containing 0.5% RiboGreen reagent was added to each well. Fluorescence intensity was measured using a Tecan microplate reader (Tecan Group Ltd., Männedorf, Switzerland), and the mRNA encapsulation efficiency (EE, %) within the LNPs was determined following the calculation below:

$$\mathrm{mRNA}\;\mathrm{EE}\;(\%)=\frac{\left(\mathrm{Triton}\;\mathrm{treated}\;\mathrm{LNP}\;\mathrm{mRNA}\;\mathrm{conc})-(\mathrm{Non}\;\mathrm{treated}\;\mathrm{LNP}\;\mathrm{mRNA}\;\mathrm{conc}\right)}{\left(\mathrm{Triton}\;\mathrm{treated}\;\mathrm{LNP}\;\mathrm{mRNA}\;\mathrm{conc}\right)}\times 100$$

For LNP physicochemical characterization, LNPs were diluted 1:20 in distilled water, and their size and polydispersity index (PDI) were measured using a Malvern Nano ZS Zetasizer (Malvern Panalytical Ltd., Malvern, UK).

### 2.3. Cell Lines

C2C12 (ATCC, CRL-1772), B16F10 (ATCC, CRL-6475), and RAW264.7 (ATCC, TIB-71) cells were purchased from ATCC (Manassas, VA, USA), incubated in high-glucose DMEM supplemented with 10% FBS at 37 °C in 5% CO2, and sub-cultured every 2 to 3 days.

### 2.4. In Vitro Assay for Luciferase Measurement and Cell Viability

C2C12, B16F10, and RAW264.7 cells were seeded in 96-well tissue culture plates (Corning-Costar, Corning, NY, USA) at a density of 4 × 10^4^ cells/mL per well. After one day, Cells were first treated with LNPs (0.1 or 0.25 μg/mL mRNA) diluted in DMEM. Subsequently, ES-2 was administered at 5, 12.5, 25, and 50 μM in 0.25% DMSO. After 4 or 20 h, all media in the wells were removed, and the attached cells were treated with lysis buffer and placed on ice for 15 min. Then, the lysates were transferred to a 96-well white plate, and the luciferase intensity of the samples was measured using the Promega^®^ Luciferase assay kit (Promega Corporation, Madison, WI, USA). For the cell viability assay for LNP and ES-2, fluorescence values in each well were measured at an absorbance of 450 nm 1 to 2 h after treatment with Cyto XTM (LPS Solution, Daejeon, Republic of Korea). For the assessment of cell viability at 48 h and 72 h, the media in the 96-well plates were removed 24 h after treatment with LNPs and ES-2, followed by washing with PBS. Fresh DMEM was then added to the wells containing the attached cells, and LNPs and ES-2 were re-administered at the same concentrations. Cell viability was subsequently measured at the indicated time points.

### 2.5. Animal Experiment

An animal experiment was conducted with approval from the Institutional Animal Care and Use Committee (IACUC) of the Catholic University of Korea (CUK) and Korea Research Institute of Bioscience and Biotechnology (KRIBB). An animal experiment was performed in accordance with the approved methods and ethical guidelines, and mice were randomly assigned to groups before the experiment.

### 2.6. In Vivo Evaluation of Luciferase Expression

C57BL/6 female mice were purchased from NaraBiotech (Seoul, Republic of Korea), and animal experiments were conducted at 8 weeks of age after acclimatization for a certain period. LNP was administered intramuscularly to mice at an mRNA dose of 6 µg (3 µg per hind leg). LNPs were mixed with ES-2 to a final concentration of 5% DMSO (*v*/*v*) before being injected into both hind legs at doses of 0.5, 1, and 2 mg/kg ES-2. Each leg received a 2.5% DMSO volume. Animals were assigned to either the control group (*n* = 3 mice) or the co-administration treatment groups (*n* = 4 mice per group). D-Luciferin was administered intraperitoneally to the mice at 6 and 24 h after the intramuscular administration of LNP and ES-2. After anesthetizing mice with isoflurane, whole-body luciferase intensity was measured using an In vivo Optical Imaging System (IVIS, PerkinElmer, Inc., Shelton, CT, USA). Relative luciferase intensity was calculated by dividing the average intensity at 24 h by the corresponding average intensity at 6 h for each group.

### 2.7. Statistical Analysis

Using GraphPad Prism version 9.5.0 (GraphPad Software, San Diego, CA, USA), graphs were generated, and statistical analyses were conducted. Statistical analyses were performed using one-way ANOVA followed by Dunnett’s multiple comparisons test or an unpaired *t* test with Welch’s correction, as appropriate. Data are presented as mean ± standard deviation (SD) and standard error of the mean (SEM), as indicated in the figure legends.

## 3. Results

### 3.1. Physicochemical Characterization of SM LNP

**Figure 2:**
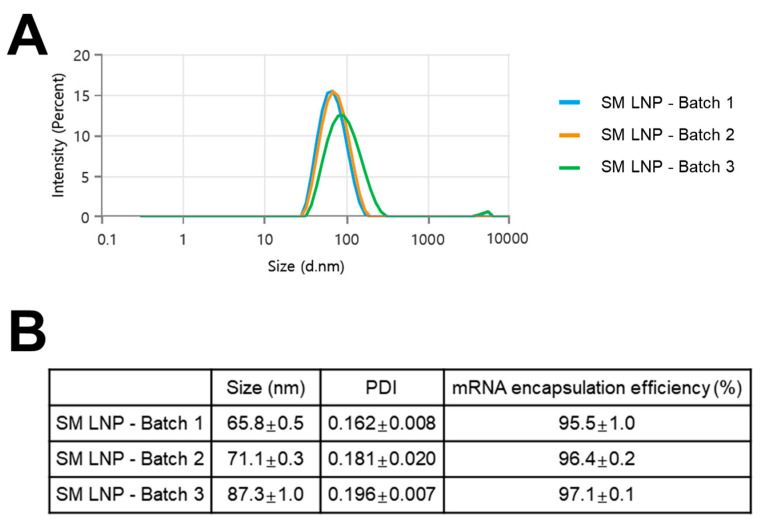
Physicochemical characterization of SM LNPs. (**A**) Representative histogram for DLS analysis of SM LNPs. (**B**) Characterization of particle size, PDI, and mRNA encapsulation efficiency (%) of SM LNPs. Data are collected from 3 independent batches of LNP preparation (*n* = 3 per batch). Data are presented as mean ± SD.

LNPs were synthesized for in vitro and in vivo experiments using SM102, DSPC, DMG-PEG, and cholesterol at a molar ratio of 43.5:10:1.5:45 [32]. Based on this formulation, the LNPs encapsulating firefly luciferase mRNA (mLuc) were prepared in triplicate (Figure 2A). DLS analysis indicates a mean size (nm) and PDI for LNP of 74.72 nm and 0.180, respectively. The average of mRNA encapsulation efficiency (%) was 96.3%. All mLuc/LNPs were synthesized with similar sizes within the 60–120 nm range, and the PDI was within 0.3 (Figure 2B). Previous studies have reported that hydrophobic or amphiphilic substances can be co-encapsulated in LNPs as a fifth component, and these inclusions endowed LNPs with novel characteristics to enhance therapeutic effects [32,33,34,35,36]. To this end, we first tried to co-encapsulate ES-2 into LNPs as the fifth component. The cholesterol component, which accounts for 45% of the total LNP composition, was replaced with ES-2 at lipid molar ratios of 5%, 10%, 15%, 20%, and 25% to prepare ES-2/LNPs (Figure S1A). The size, PDI, and mRNA encapsulation efficiency of the ES-2-containing LNPs are similar to the values observed in conventional LNPs (Figure S1B). However, all ES-2 containing LNPs exhibited decreased mRNA expression levels in the cell lines, C2C12 and B16F10, compared to the control SM LNP (Figure S1C). Overall, the co-treatment strategy of ES-2 with LNPs, rather than the co-encapsulation, was chosen.

### 3.2. Co-Treatment of LNPs with ES-2 Enhances mRNA Delivery Efficiency in Vitro

**Figure 3:**
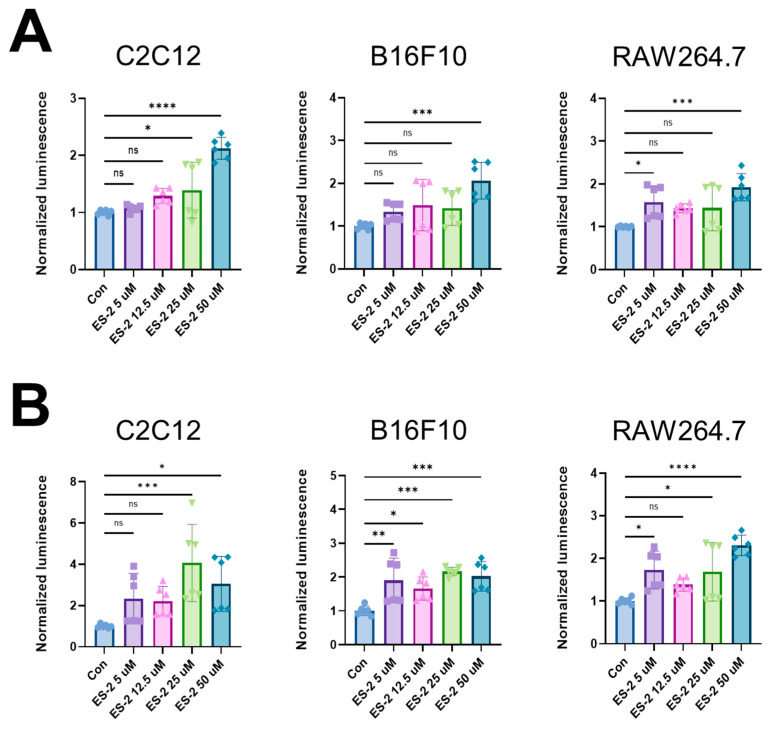
Evaluation of in vitro delivery efficiency following co-treatment of SM LNPs and ES-2. (**A**,**B**) C2C12, B16F10, and RAW264.7 cells were co-treated with SM LNPs and ES-2. mRNA concentration of 0.1 μg/mL (**A**) and 0.25 μg/mL (**B**). Luminescence was measured at 20 h post-treatment and normalized to the control group (sole LNP treatment). Data are collected from two independent experiments (*n* = 3 per experiment) and presented as mean ± SD. Statistical analysis was performed using one-way ANOVA followed by Dunnett’s multiple comparisons test compared to the control. Statistical significance is represented as: Non-significant (ns), (*) *p* < 0.05, (**) *p* < 0.01, (***) *p* < 0.001, (****) *p* < 0.0001.

To investigate whether co-treatment with ES-2 influences LNP-mediated mRNA delivery across different cell types, three cell lines (C2C12, B16F10, and RAW264.7) were treated with LNPs at mRNA concentrations of 0.1 μg/mL and 0.25 μg/mL in the presence of ES-2 at 5 μM, 12.5 μM, 25 μM, and 50 μM. At 4 h post-treatment, a significant increase in mRNA expression was observed in C2C12 cells by the ES-2 co-treatment, but not in B16F10 cells. Notably, in C2C12 cells, LNP-mediated mRNA delivery efficiency increased by up to 3.05-fold and 2.97-fold at mRNA doses of 0.1 μg/mL and 0.25 μg/mL, respectively, in the presence of ES-2 compared to the control group (only LNP) (Figure S2A,B). At 20 h post-treatment, ES-2 co-treatment significantly enhanced mRNA expression across all cell lines compared to the sole LNP-treated control group. At mRNA doses of 0.1 μg/mL and 0.25 μg/mL, the maximum increases in mRNA expression reached 2.06-fold and 2.16-fold in B16F10 cells and 1.92-fold and 2.31-fold in RAW264.7 cells, respectively. Similarly, in C2C12 cells, the maximum increases in mRNA expression reached 2.13-fold and 4.06-fold at mRNA doses of 0.1 μg/mL and 0.25 μg/mL, respectively (Figure 3A,B).

### 3.3. In Vitro Cytotoxicity Evaluation of the ES-2 Co-Treatment

**Figure 4:**
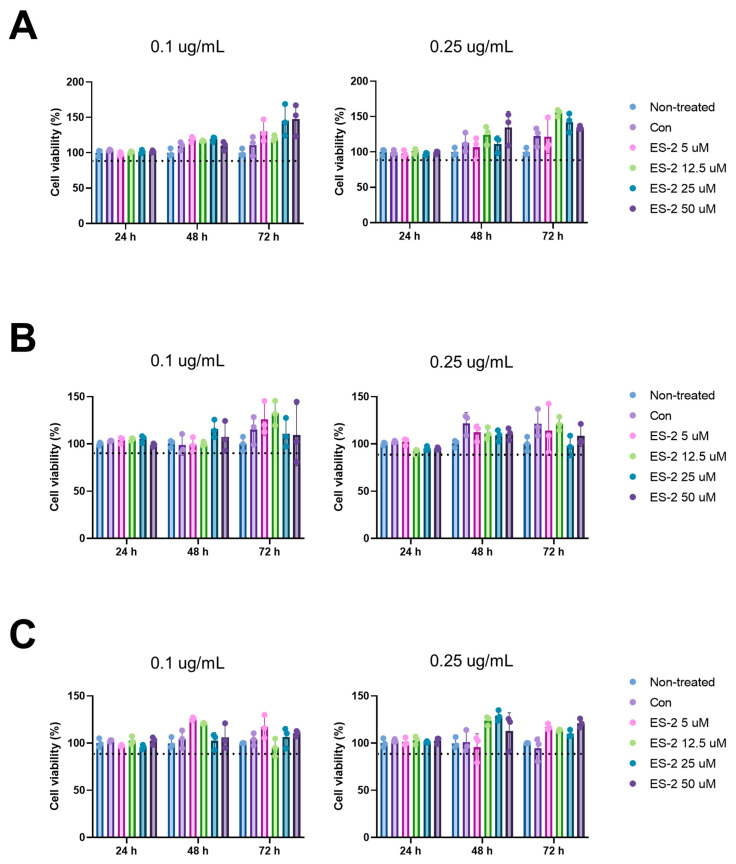
In vitro cytotoxicity test following co-treatment of SM LNPs with ES-2. (**A**) C2C12, (**B**) B16F10, and (**C**) RAW264.7 cells were co-treated with mRNA/LNP and ES-2, and cell viability was measured (*n* = 3). Data are presented as mean ± SD. The black dashed line indicates the 90% threshold.

In previous studies, ES-2 has been used in the experiments of plant and animal cells at various concentrations ranging from 4 μM to 250 μM [27,29,30]. To assess the potential toxicity of the co-treatment, 3 different cell lines were co-treated with LNPs and ES-2, and cell viability was then measured. As shown in the results, more than 90% cell viability was maintained across all groups in the tested cell lines at 24, 48, and 72 h, indicating no apparent cytotoxicity following co-treatment with LNPs and ES-2 (Figure 4A–C). These results suggest that co-treatment of ES-2 and LNP has no significant toxic effect at the indicated doses in vitro.

### 3.4. Stability of LNPs Following Mixing with ES-2

**Figure 5:**
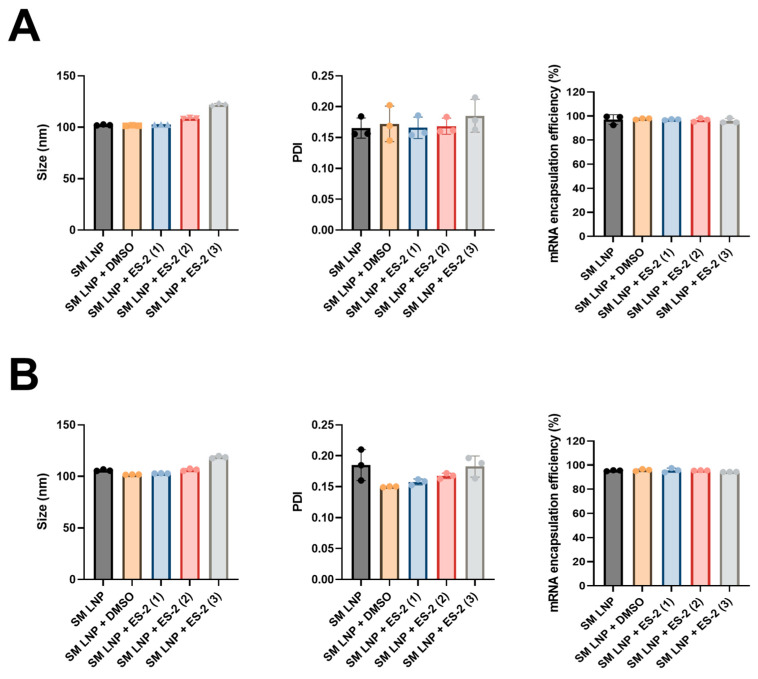
Assessment of LNP integrity after mixing with ES-2. The physicochemical properties of LNPs, including particle size, PDI, and mRNA encapsulation efficiency (%), were evaluated following the addition of ES-2. Measurements were performed after (**A**) incubation with DMSO or ES-2 for 30 min at room temperature and (**B**) subsequent storage at 4 °C for 24 h (*n* = 3). Data are presented as mean ± SD.

Prior to in vivo administration, the stability of LNPs after mixing with ES-2 was evaluated. LNPs were mixed with ES-2, and their size, PDI, and mRNA encapsulation efficiency (%) were assessed after 30 min at room temperature and again after 24 h of storage at 4 °C. For this analysis, SM LNP was used without modification, whereas SM LNP + DMSO was formulated with 5% DMSO as the vehicle control. In addition, SM LNP + ES-2 (1), SM LNP + ES-2 (2), and SM LNP + ES-2 (3) were formulated to contain ES-2 at doses corresponding to 0.5, 1, and 2 mg/kg, respectively, while maintaining the same DMSO content as that of the SM LNP + DMSO group. No significant differences in physicochemical properties were observed among all groups (Figure 5A,B). These results indicate that the physicochemical properties of the LNPs were stably maintained even after mixing with ES-2 dissolved in DMSO.

### 3.5. Co-Administration of LNPs with ES-2 Enhances the in Vivo mRNA Delivery Efficiency

**Figure 6:**
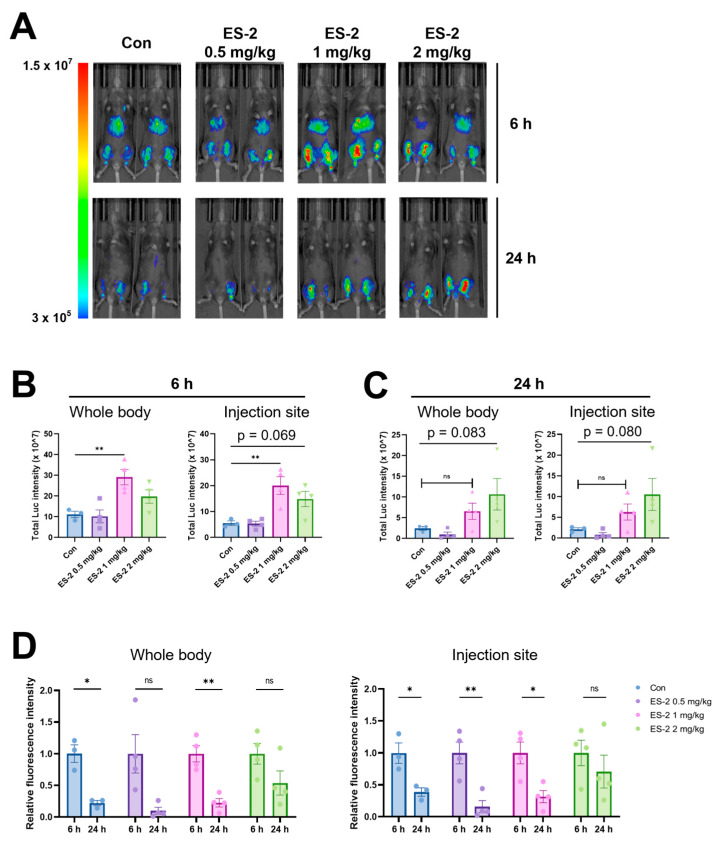
In vivo evaluation of mRNA delivery efficiency following co-administration of SM LNPs with ES-2. (**A**) Representative in vivo bioluminescence images of mRNA delivery efficiency following co-administration of SM LNP with ES-2. Luciferase intensity was measured using an IVIS imaging system at 6 h and 24 h post-injection. (**B**) Luminescence intensity at 6 h post-injection. (**C**) Luminescence intensity at 24 h post-injection. (**D**) Relative fluorescence intensity at 6 h and 24 h post-injection (*n* = 3–4 mice/group). Data are presented as mean ± SEM. Statistical analysis for (**B**) and (**C**) was performed using one-way ANOVA followed by Dunnett’s multiple comparisons test compared to the control. Statistical analysis for (**D**) was performed using an unpaired *t*-test with Welch’s correction. Statistical significance is represented as: Non-significant (ns), (*) *p* < 0.05, (**) *p* < 0.01.

In a previous study, ES-2 was co-administered intraperitoneally to enhance the anticancer effect of cisplatin [28]. Based on that study, LNPs were co-administered with ES-2 at various concentrations of 0.5 mg/kg, 1 mg/kg, and 2 mg/kg. As shown, LNPs and ES-2 were simultaneously injected through the hind leg muscle, and a significant difference in the mRNA expression was observed between the groups (Figure 6A). Notably, at 6 h, the group receiving 1 and 2 mg/kg of ES-2 exhibited enhanced mRNA delivery compared to the sole LNP-treated control group in both the whole body and the injection site. The co-administered group of ES-2 1 mg/kg dose exhibited the highest mRNA delivery effect at 6 h post-injection, which is a 2.62-fold increase of mRNA expression at the whole-body level and a 3.63-fold increase at the injection site compared to the control group. The co-administration group of 2 mg/kg dose resulted in a 2.68-fold increase in mRNA delivery at the injection site at 6 h post-administration, although this effect did not reach statistical significance (*p* = 0.069) (Figure 6B). At 24 h post-injection, co-administration of LNP with ES-2 dose of 2 mg/kg resulted in a 4.37-fold increase in whole-body mRNA expression (*p* = 0.083) and a 4.91-fold increase at the injection site (*p* = 0.080) compared to the sole LNP-treated control group (Figure 6C). The lower dose of ES-2 (0.5 mg/kg) did not induce an increase in the mRNA delivery at both 6 and 24 h post-administration compared to the control group (Figure 6B,C). In addition, the ES-2 2 mg/kg co-administration group exhibited a prolonged in vivo mRNA delivery effect compared with other groups (Figure 6D). In conclusion, co-administration of LNP with ES-2 enhances and prolongs the LNP-mediated mRNA delivery, reaching up to a 2 to 4-fold increase in vivo dependent ES-2 dose.

## 4. Discussion

Previous studies have demonstrated that modulation of intracellular trafficking pathways can enhance LNP-mediated RNA delivery. For example, inhibition of NPC1 increased cytosolic siRNA accumulation by up to 15-fold in MEFs [15]. In another study, co-treatment with trafficking inhibitors such as NAV2729 or ES-5 enhanced mRNA delivery efficiency by approximately 1.5–2-fold in vitro and 1.3-fold in vivo [23]. Previous mechanistic studies reported that the inhibition of Exo70 suppresses exocytosis and induces intracellular endosome accumulation [27,30]. However, the effect of Exo70-inhibition on mRNA/LNP delivery has not been studied yet. To this end, we hypothesized that transient inhibition of Exo70 by ES-2 could prolong intracellular retention of LNPs and improve mRNA delivery efficiency. Consistent with this hypothesis, co-administration of a specific concentration of ES-2 with LNPs significantly increased delivery efficiency both in vitro and in vivo. These findings suggest that the inhibition of Exo70 may represent a useful strategy to enhance and prolong LNP-mediated mRNA expression.

The incorporation of a fifth component into LNP formulations has been reported to alter physicochemical properties—such as particle size, PDI, surface charge, and mRNA encapsulation efficiency—as well as delivery efficiency, depending on the molar ratio of a fifth component [33,34,35]. In this study, cholesterol was partially substituted with ES-2 to evaluate its synergistic potential. Although the resulting formulations exhibited physicochemical characteristics comparable to those of conventional LNPs, reduced delivery efficiency was observed in certain ES-2-incorporated groups. While the precise mechanism underlying this reduction remains to be fully resolved, it may be attributed to excessive intracellular ES-2 activity or overly strong interactions between ES-2 and the lipid components. Such interactions could potentially interfere with critical trafficking processes, including membrane fusion and endosomal escape.

Elucidating the detailed mechanisms underlying the structure–activity relationship of LNP formulations remains a major challenge. In the case of ES-2, its unique structural characteristics may interfere with the intrinsic delivery efficiency of LNPs when directly incorporated into the formulation. In particular, because ES-2 contains fluorine (F)- and iodine (I)-containing moieties [27], predicting how interactions between ES-2 and LNP components influence the physicochemical properties and delivery behavior of the nanoparticles during formulation remains difficult.

Consistent with a previous report demonstrating heterogeneous cellular responses to LNP-mediated mRNA delivery [36], the differential enhancement by ES-2 observed at 4 h in two cell lines (C2C12 and B16F10) may reflect cell-type-dependent differences in intracellular trafficking, endosomal processing, or sensitivity to exocyst complex inhibition. In contrast, statistically significant enhancement was observed at 24 h, suggesting that the effect of ES-2 on LNP-mediated delivery became more broadly evident over time.

Further research is needed to determine why the specific concentration of ES-2, when mixed with LNPs immediately prior to intramuscular injection, differentially influences delivery efficiency and the duration of expression. Understanding the underlying mechanisms behind these dose-dependent effects will be crucial for optimizing the synergistic potential of ES-2 and LNP co-administration.

## 5. Conclusions

In this study, we employed ES-2, an Exo70 inhibitor, as an adjuvant for mRNA/LNP delivery and evaluated its beneficial effects on mRNA delivery both in vitro and in vivo. Co-treatment of LNPs with ES-2 enhanced LNP-mediated mRNA delivery by up to 4.06-fold in vitro. Consistent with the in vitro findings, co-administration of LNPs with ES-2 significantly improved in vivo mRNA delivery efficiency by 3.63-fold at an ES-2 dose of 1 mg/kg at 6 h post-injection. Furthermore, at an ES-2 dose of 2 mg/kg with LNPs, the decrease in mRNA expression from 6 to 24 h post-injection was significantly attenuated, indicating prolonged mRNA expression. Collectively, these findings suggest that Exo70 and exocyst complex inhibitors may serve as promising adjuvants for the development of mRNA/LNP therapeutics.

## Data Availability

The data that support the findings of this study are available from the corresponding author upon reasonable request.

## Supplementary Materials

The following supporting information can be downloaded at: https://www.mdpi.com/article/10.3390/pharmaceutics18060650/s1, Figure S1. Preparation of ES-2–incorporated LNPs by adjusting cholesterol ratios. Figure S2. In vitro mRNA delivery efficiency of SM LNPs in the presence of ES-2.

## Abbreviations

LNP (Lipid nanoparticle), MEFs (Mouse embryonic fibroblasts), mRNA (Messenger RNA), siRNA (Small interfering RNA), shRNA (Short hairpin RNA), ES-2 (Endosidin-2), MVE (Multivesicular endosome), PDI (Polydispersity index), EE (Encapsulation efficiency).

## References

[1] Hou X., Zaks T., Langer R., Dong Y. (2021). Lipid nanoparticles for mRNA delivery. Nat. Rev. Mater..

[2] McKay P.F., Hu K., Blakney A.K., Samnuan K., Brown J.C., Penn R., Zhou J., Bouton C.R., Rogers P., Polra K. (2020). Self-amplifying RNA SARS-CoV-2 lipid nanoparticle vaccine candidate induces high neutralizing antibody titers in mice. Nat. Commun..

[3] Polack F.P., Thomas S.J., Kitchin N., Absalon J., Gurtman A., Lockhart S., Perez J.L., Perez Marc G., Moreira E.D., Zerbini C. (2020). Safety and Efficacy of the BNT162b2 mRNA Covid-19 Vaccine. N. Engl. J. Med..

[4] Tenchov R., Bird R., Curtze A.E., Zhou Q. (2021). Lipid Nanoparticles horizontal line From Liposomes to mRNA Vaccine Delivery, a Landscape of Research Diversity and Advancement. ACS Nano.

[5] Chatterjee S., Kon E., Sharma P., Peer D. (2024). Endosomal escape: A bottleneck for LNP-mediated therapeutics. Proc. Natl. Acad. Sci. USA.

[6] Cullis P.R., Felgner P.L. (2024). The 60-year evolution of lipid nanoparticles for nucleic acid delivery. Nat. Rev. Drug Discov..

[7] Jozic A., Le Roux C., Kim J., Berchel M., Sahel D.K., Bodi E.K., Palumbo M., Vasudevan A., Murthy N.T.V., Eygeris Y. (2026). In vivo endosomal escape assay identifies mechanisms for efficient hepatic LNP delivery. Nat. Biotechnol..

[8] Wei P.S., Thota N., John G., Chang E., Lee S., Wang Y., Ma Z., Tsai Y.H., Mei K.C. (2024). Enhancing RNA-lipid nanoparticle delivery: Organ- and cell-specificity and barcoding strategies. J. Control. Release.

[9] Hosseini-Kharat M., Bremmell K.E., Prestidge C.A. (2025). Why do lipid nanoparticles target the liver? Understanding of biodistribution and liver-specific tropism. Mol. Ther. Methods Clin. Dev..

[10] Luzio J.P., Pryor P.R., Bright N.A. (2007). Lysosomes: Fusion and function. Nat. Rev. Mol. Cell Biol..

[11] Radulovic M., Yang C., Stenmark H. (2026). Lysosomal membrane homeostasis and its importance in physiology and disease. Nat. Rev. Mol. Cell Biol..

[12] Gilleron J., Querbes W., Zeigerer A., Borodovsky A., Marsico G., Schubert U., Manygoats K., Seifert S., Andree C., Stoter M. (2013). Image-based analysis of lipid nanoparticle-mediated siRNA delivery, intracellular trafficking and endosomal escape. Nat. Biotechnol..

[13] Bai Y., Cui L., Liu T., Wang Z., Cao H., He S., Qiu J., Li Y., Zhou Y., Shi J. (2025). Recent Advances in mRNA-LNP Delivery Systems for Extrahepatic Organs: A Review. Mol. Pharm..

[14] Zhang H., Meng C., Yi X., Han J., Wang J., Liu F., Ling Q., Li H., Gu Z. (2024). Fluorinated Lipid Nanoparticles for Enhancing mRNA Delivery Efficiency. ACS Nano.

[15] Sahay G., Querbes W., Alabi C., Eltoukhy A., Sarkar S., Zurenko C., Karagiannis E., Love K., Chen D., Zoncu R. (2013). Efficiency of siRNA delivery by lipid nanoparticles is limited by endocytic recycling. Nat. Biotechnol..

[16] Wang J., Chen R., Xie Y., Qin X., Zhou Y., Xu C. (2025). Endo/Lysosomal-Escapable Lipid Nanoparticle Platforms for Enhancing mRNA Delivery in Cancer Therapy. Pharmaceutics.

[17] Bar-On L., Cohen H., Elia U., Cherry-Mimran L., Cohen O., Erez N. (2025). The mRNA component of LNP-mRNA vaccines triggers IFNAR-dependent immune activation which attenuates the adaptive immune response. Front. Immunol..

[18] Lobb T.A., Dickson A., Guo W., Beeram S., Carrero J.A., Dalben Y., DiPaolo R.J., Alspach E., Tse L.V., Ferris S.T. (2026). Type I interferon restricts mRNA vaccine efficacy through suppression of antigen uptake in cDCs. npj Vaccines.

[19] Lokugamage M.P., Gan Z., Zurla C., Levin J., Islam F.Z., Kalathoor S., Sato M., Sago C.D., Santangelo P.J., Dahlman J.E. (2020). Mild Innate Immune Activation Overrides Efficient Nanoparticle-Mediated RNA Delivery. Adv. Mater..

[20] Grau M., Wagner E. (2024). Strategies and mechanisms for endosomal escape of therapeutic nucleic acids. Curr. Opin. Chem. Biol..

[21] Cavegn A., Waldner S., Wang D., Sedzicki J., Kuzucu E.U., Zogg M., Lotter C., Huwyler J. (2025). Intracellular processing of DNA-lipid nanoparticles: A quantitative assessment by image segmentation. J. Control. Release.

[22] Suzuki Y., Katsurada Y., Hyodo K. (2023). Differences and Similarities of the Intravenously Administered Lipid Nanoparticles in Three Clinical Trials: Potential Linkage between Lipid Nanoparticles and Extracellular Vesicles. Mol. Pharm..

[23] Shin J., Douglas C.J., Zhang S., Seath C.P., Bao H. (2024). Targeting Recycling Endosomes to Potentiate mRNA Lipid Nanoparticles. Nano Lett..

[24] Ren J., Guo W. (2012). ERK1/2 regulate exocytosis through direct phosphorylation of the exocyst component Exo70. Dev. Cell.

[25] Sekeres J., Pejchar P., Santrucek J., Vukasinovic N., Zarsky V., Potocky M. (2017). Analysis of Exocyst Subunit EXO70 Family Reveals Distinct Membrane Polar Domains in Tobacco Pollen Tubes. Plant Physiol..

[26] Zuo X., Zhang J., Zhang Y., Hsu S.C., Zhou D., Guo W. (2006). Exo70 interacts with the Arp2/3 complex and regulates cell migration. Nat. Cell Biol..

[27] Zhang C., Brown M.Q., van de Ven W., Zhang Z.M., Wu B., Young M.C., Synek L., Borchardt D., Harrison R., Pan S. (2016). Endosidin2 targets conserved exocyst complex subunit EXO70 to inhibit exocytosis. Proc. Natl. Acad. Sci. USA.

[28] Zhao Y., Hong X., Chen X., Hu C., Lu W., Xie B., Zhong L., Zhang W., Cao H., Chen B. (2021). Deregulation of Exo70 Facilitates Innate and Acquired Cisplatin Resistance in Epithelial Ovarian Cancer by Promoting Cisplatin Efflux. Cancers.

[29] Yao Y., Subedi K., Liu T., Khalasawi N., Pretto-Kernahan C.D., Wotring J.W., Wang J., Yin C., Jiang A., Fu C. (2022). Surface translocation of ACE2 and TMPRSS2 upon TLR4/7/8 activation is required for SARS-CoV-2 infection in circulating monocytes. Cell Discov..

[30] Liu D.A., Tao K., Wu B., Yu Z., Szczepaniak M., Rames M., Yang C., Svitkina T., Zhu Y., Xu F. (2023). A phosphoinositide switch mediates exocyst recruitment to multivesicular endosomes for exosome secretion. Nat. Commun..

[31] Bai S., Hou W., Yao Y., Meng J., Wei Y., Hu F., Hu X., Wu J., Zhang N., Xu R. (2022). Exocyst controls exosome biogenesis via Rab11a. Mol. Ther. Nucleic Acids.

[32] Yong S.-B., Park O.H., Cho S. (2024). Microbiome-Derived Lipid Nanoparticles for Improved Immunogenicity of mRNA Vaccines. ACS Mater. Lett..

[33] Patel S., Ashwanikumar N., Robinson E., Xia Y., Mihai C., Griffith J.P., Hou S., Esposito A.A., Ketova T., Welsher K. (2020). Naturally-occurring cholesterol analogues in lipid nanoparticles induce polymorphic shape and enhance intracellular delivery of mRNA. Nat. Commun..

[34] Bogaert B., Debisschop A., Ehouarne T., Van Eeckhoutte H.P., De Volder J., Jacobs A., Pottie E., De Rycke R., Crabbe A., Mestdagh P. (2024). Selective Replacement of Cholesterol with Cationic Amphiphilic Drugs Enables the Design of Lipid Nanoparticles with Improved RNA Delivery. Nano Lett..

[35] Isaac I., Patel L., Tran N., Singam A., Yun D., Guha P., Park S., Bhattacharya C. (2025). Reengineering Endogenous Targeting Lipid Nanoparticles (ENDO) for Systemic Delivery of mRNA to Pancreas. Adv. Mater..

[36] Dobrowolski C., Paunovska K., Schrader Echeverri E., Loughrey D., Da Silva Sanchez A.J., Ni H., Hatit M.Z.C., Lokugamage M.P., Kuzminich Y., Peck H.E. (2022). Nanoparticle single-cell multiomic readouts reveal that cell heterogeneity influences lipid nanoparticle-mediated messenger RNA delivery. Nat. Nanotechnol..

