# Supplementary material for: Inhibition of Exo70, an Exocyst Complex Component, Enhances mRNA Delivery Efficiency of Lipid Nanoparticle
Source: Pharmaceutics. 2026 May 25;18(6):650. doi: 10.3390/pharmaceutics18060650 (PMC13306191; doi:10.3390/pharmaceutics18060650)
Supplement: Supplementary file 1 [file pharmaceutics-18-00650-s001.zip › pharmaceutics-4275261-supplementary.pdf]

Supplementary Information.

Inhibition of Exo70, an Exocyst Complex Component, Enhances mRNA Delivery Efficiency of Lipid Nanoparticle

Minki Ha[1], *Seok-Beom Yong*[1]*

[1] Department of Medical and Biological Sciences, The Catholic University of Korea, Jibongro 43, Bucheon-si, Gyeonggi-do 14662, Republic of Korea

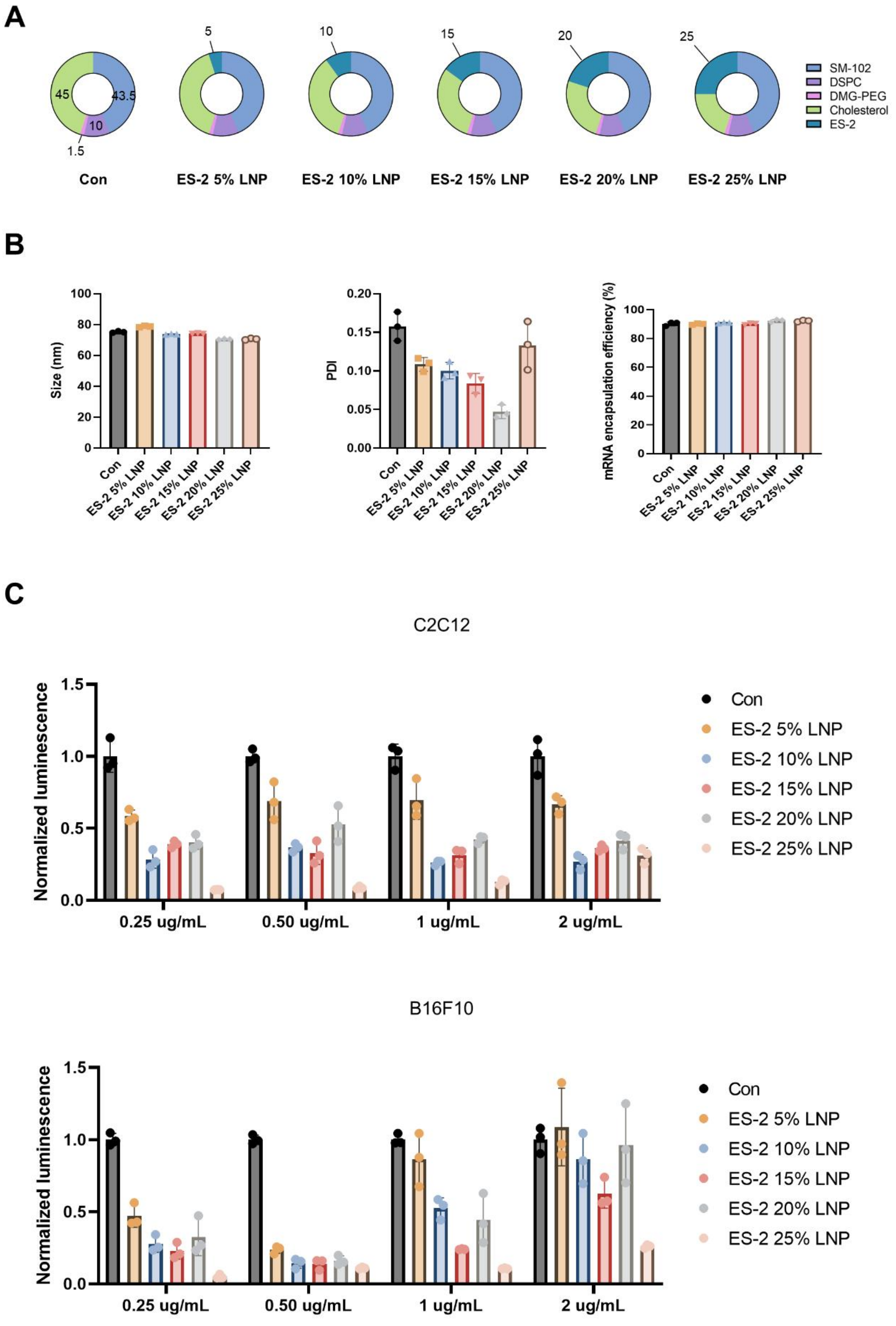

Figure S1. Preparation of ES-2–incorporated LNPs by adjusting cholesterol ratios. (A) Molar ratios of LNP components formulated by replacing cholesterol with ES-2. (B) Size, PDI, and mRNA encapsulation efficiency (%) of ES-2–incorporated LNPs. (C) Evaluation of mRNA delivery efficiency of ES-2–incorporated LNPs (n=3). Data are presented as mean ± SD.

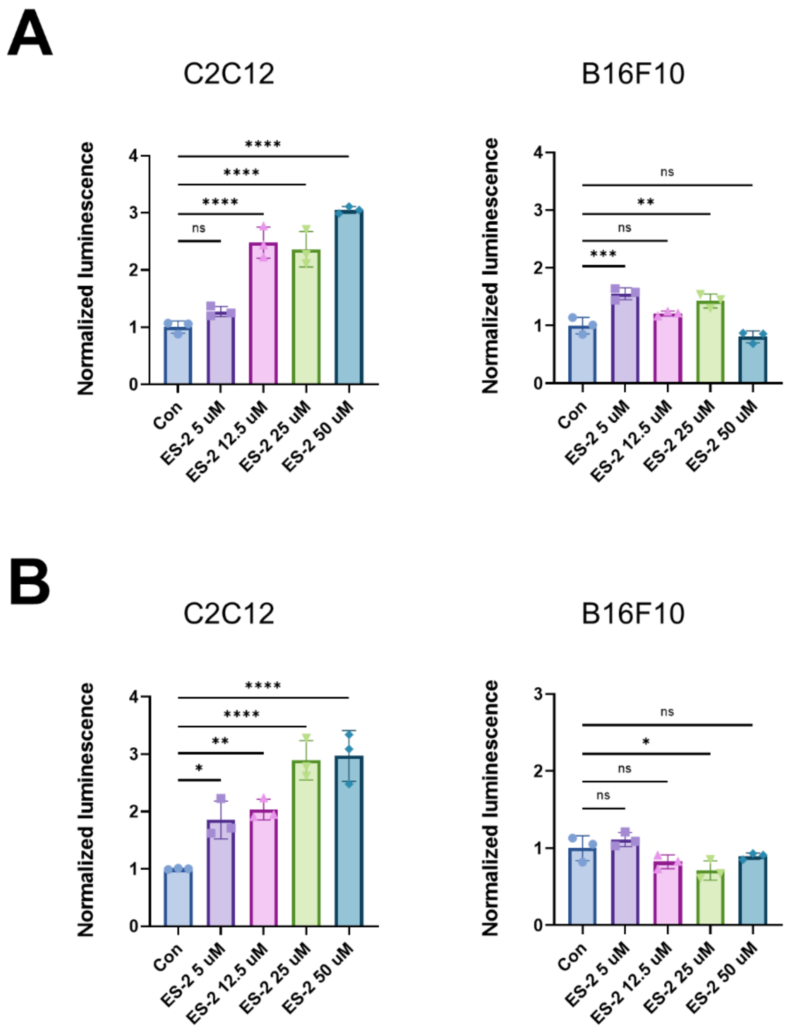

Figure S2. ***In vitro*** **mRNA delivery efficiency of SM LNPs in the presence of ES-2.** (A, B) C2C12 and B16F10 cells were co-treated with SM LNPs and ES-2 at mRNA concentrations of (A) 0.1 µg/mL, and (B) 0.25 µg/mL. Luminescence was measured at 4 h post-treatment and normalized to the value of sole mRNA/LNP-treated control (n=3). Data are presented as mean ± SD. Statistical analysis was performed using one-way ANOVA followed by Dunnett's multiple comparisons test compared to the control. Statistical significance is represented as: Non-significant (ns), (*) $p < 0.05$, (**) $p < 0.01$, (***) $p < 0.001$, (****) $p < 0.0001$.
